# 1-[5-Acetyl-2,6-dimethyl-4-(5-phenyl-1*H*-pyrazol-3-yl)-1,4-dihydro­pyridin-3-yl]ethanone monohydrate

**DOI:** 10.1107/S1600536812042614

**Published:** 2012-10-20

**Authors:** Arun M. Islor, Shridhar Malladi, S. Sundershan, Thomas Gerber, Eric Hosten, Richard Betz

**Affiliations:** a National Institute of Technology-Karnataka, Department of Chemistry, Organic Chemistry Laboratory, Surathkal, Mangalore 575 025, India; b UAS Hebbal, Veterinary College, Department of Microbiology, Bangalore 24, India; c Nelson Mandela Metropolitan University, Summerstrand Campus, Department of Chemistry, University Way, Summerstrand, PO Box 77000, Port Elizabeth, 6031, South Africa

## Abstract

In the title compound, C_20_H_21_N_3_O_2_·H_2_O, the aza-substitued six-membered ring adopts a ^L4^ *B* conformation. In the crystal, classical N—H⋯O, N—H⋯N and O—H⋯O hydrogen bonds connect the entities into a three-dimensional network. Intra­molecular C—H⋯O contacts are also observed.

## Related literature

For the pharmaceutical properties of 1,4-dihydro­pyridine-derived drugs, see: Janis & Triggle (1983 ▶); Boecker & Guengerich (1986 ▶); Gordeev *et al.* (1996 ▶); Buhler & Kiowski (1987 ▶); Vo *et al.* (1995 ▶). For the conformational analysis of puckering factors of five- and six-membered rings, see: Cremer & Pople (1975 ▶). For graph-set analysis of hydrogen bonds, see: Etter *et al.* (1990 ▶); Bernstein *et al.* (1995 ▶).
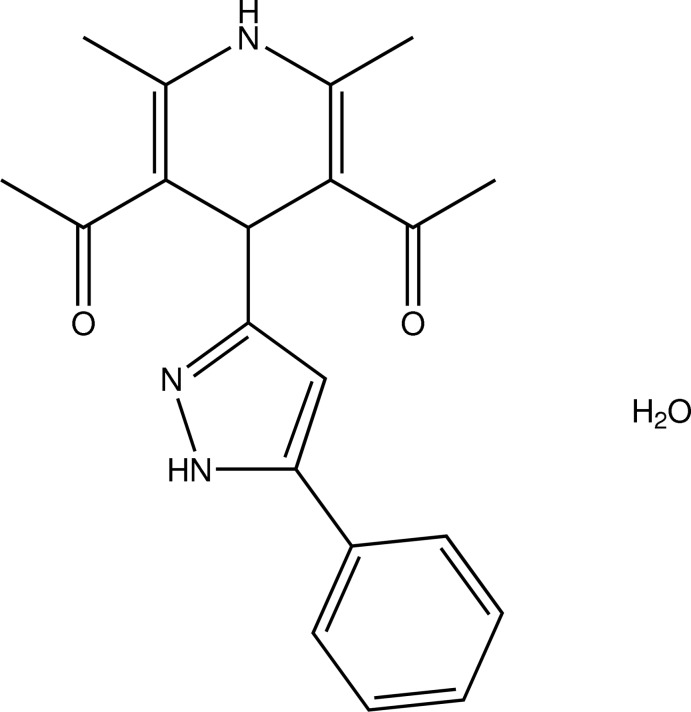


## Experimental

### 

#### Crystal data

C_20_H_21_N_3_O_2_·H_2_O
*M* *_r_* = 353.41
Monoclinic, 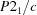

*a* = 10.3516 (2) Å
*b* = 12.4352 (3) Å
*c* = 15.4101 (3) Å
β = 112.798 (1)°
*V* = 1828.68 (7) Å^3^
*Z* = 4
Mo *K*α radiation
μ = 0.09 mm^−1^
*T* = 200 K
0.41 × 0.34 × 0.18 mm

#### Data collection

Bruker APEXII CCD diffractometer
Absorption correction: multi-scan (*SADABS*; Bruker, 2008 ▶) *T*_min_ = 0.965, *T*_max_ = 0.984
17882 measured reflections
4559 independent reflections
3849 reflections with *I* > 2σ(*I*)
*R*_int_ = 0.015

#### Refinement

*R*[*F* ^2^ > 2σ(*F* ^2^)] = 0.040
*wR*(*F* ^2^) = 0.114
*S* = 1.04
4559 reflections
255 parameters
H atoms treated by a mixture of independent and constrained refinement
Δρ_max_ = 0.30 e Å^−3^
Δρ_min_ = −0.21 e Å^−3^

### 

Data collection: *APEX2* (Bruker, 2010 ▶); cell refinement: *SAINT* (Bruker, 2010 ▶); data reduction: *SAINT*; program(s) used to solve structure: *SHELXS97* (Sheldrick, 2008 ▶); program(s) used to refine structure: *SHELXL97* (Sheldrick, 2008 ▶); molecular graphics: *ORTEPIII* (Farrugia, 1997 ▶) and *Mercury* (Macrae *et al.*, 2008 ▶); software used to prepare material for publication: *SHELXL97* and *PLATON* (Spek, 2009 ▶).

## Supplementary Material

Crystal structure: contains datablock(s) I, global. DOI: 10.1107/S1600536812042614/hg5260sup1.cif

Supplementary material file. DOI: 10.1107/S1600536812042614/hg5260Isup2.cdx

Structure factors: contains datablock(s) I. DOI: 10.1107/S1600536812042614/hg5260Isup3.hkl

Supplementary material file. DOI: 10.1107/S1600536812042614/hg5260Isup4.cml

Additional supplementary materials:  crystallographic information; 3D view; checkCIF report

## Figures and Tables

**Table 1:** Hydrogen-bond geometry (Å, °)

*D*—H⋯*A*	*D*—H	H⋯*A*	*D*⋯*A*	*D*—H⋯*A*
N2—H2*A*⋯O3^i^	0.938 (19)	1.855 (19)	2.7822 (14)	169.1 (16)
N3—H3⋯N1^ii^	0.878 (17)	2.075 (17)	2.9454 (13)	171.1 (15)
O3—H3*B*⋯O2^iii^	0.86 (2)	1.92 (2)	2.7838 (13)	175.8 (17)
O3—H3*C*⋯O1^iv^	0.87 (2)	1.88 (2)	2.7372 (14)	169.8 (18)
C6—H6⋯O2	0.95	2.44	3.282 (2)	148
C10—H10⋯O2	1.00	2.33	2.7624 (13)	105

Symmetry codes: (i) 
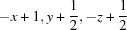
; (ii) 
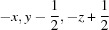
; (iii) 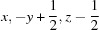; (iv) 
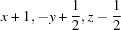
.

**Fig. 1.**
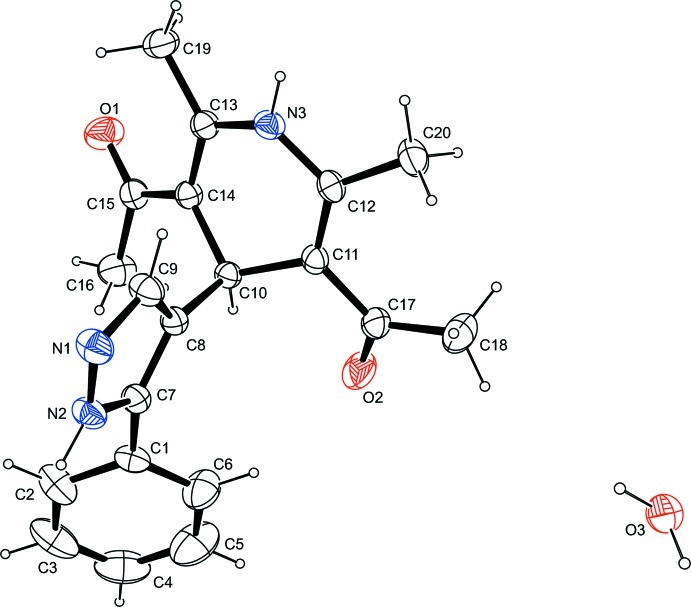
The molecular structure of the title compound, with atom labels and anisotropic displacement ellipsoids (drawn at 50% probability level).

**Fig. 2.**
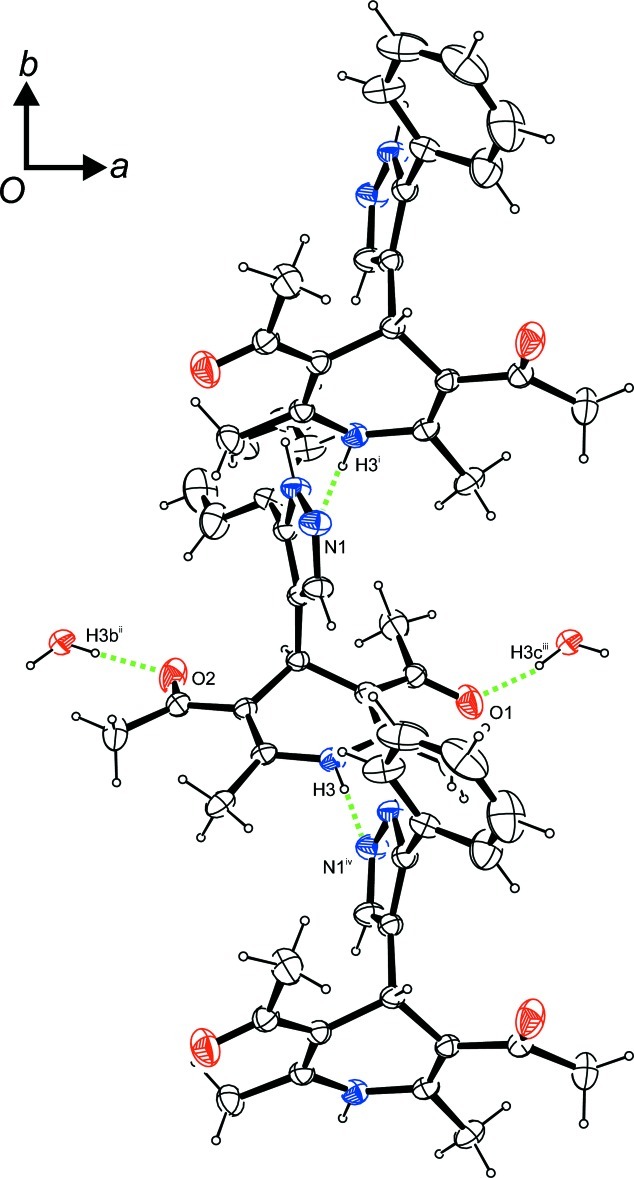
Intermolecular contacts, viewed along [0 0 - 1]. For clarity, only a selection of contacts apparent in the crystal structure is depicted. Symmetry operators: i -x, y + 1/2, -z + 1/2; iix, -y + 1/2, z + 1/2; iiix - 1, -y + 1/2, z + 1/2; iv -x, y - 1/2, -z + 1/2.

**Fig. 3.**
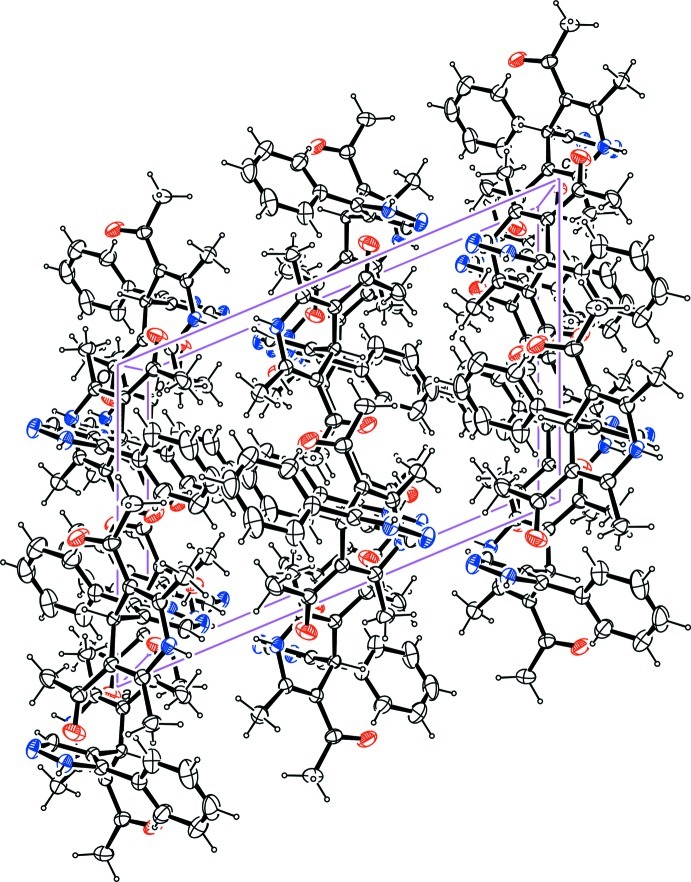
Molecular packing of the title compound, viewed along [0 1 0] (anisotropic displacement ellipsoids drawn at 50% probability level).

## supplementary crystallographic information

### Comment

In recent years, considerable attention has been paid to the synthesis of 1,4-dihydropyridines owing to their significant biological activity. 1,4-Dihydropyridine-containing drugs (1,4-DHPs), such as nifedipine, nicardipine, amlodipine, felodipine and others have been found to be useful as calcium channel blockers (Janis & Triggle 1983; Boecker & Guengerich 1986; Gordeev *et al.*, 1996) and are used most frequently as cardiovascular agents for the treatment of hypertension (Buhler & Kiowski 1987). A number of DHP derivatives are employed as potential drug candidates for the treatment of congestive heart failure (Vo *et al.*, 1995). Prompted by the diverse activities of 1,4-Dihydropyridines, we have synthesized the title compound to study its crystal structure.

The molecule features a pyrazole core bearing a phenyl as well as a 1,4-dihydropyridine-derived substituent. The six-membered ring of the latter adopts a ^L4^*B* conformation according to a puckering analysis (Cremer & Pople, 1975). The least-squares planes defined by the respective intracyclic atoms of the phenyl group as well as the 1,4-dihydropyridine core enclose angles of 52.79 (8) ° and 88.10 (7) ° with the least-squares plane defined by the non-hydrogen atoms of the central pyrazole core (Fig. 1).

In the crystal, classical hydrogen bonds of the O–H···O, N–H···O and N–H···N type are apparent involving both ketonic oxygen atoms and the non-protonated nitrogen atom as acceptors. In addition, two intramolecular C–H···O contacts can be observed whose range falls by more than 0.2 Å below the sum of van-der-Waals radii of the atoms participating. The latter stem from a hydrogen atom on the phenyl group as well as the methine group and have the same ketonic oxygen atom as acceptor. In total, the entities of the title compound are connected to a three-dimensional network in the crystal structure. In terms of graph-set analysis (Etter *et al.*, 1990; Bernstein *et al.*, 1995), the descriptor for these contacts is *S*(5)*S*(9)*DDDC*^1^_1_(8) on the unary level. Metrical parameters as well as information about the symmetry of these contacts are summarized in Table 1. The shortest intercentroid distance between two aromatic systems was measured at 4.7199 (10) Å and is apparent between the pyrazol and the phenyl moiety in neighbouring molecules (Fig. 2).

The packing of the title compound in the crystal structure is shown in Figure 3.

### Experimental

3-phenyl-1*H*-pyrazole-4-carbaldehyde (0.172 g, 1.0 mmol), acetylacetone (0.2 g, 2.0 mmol) and ammonium acetate (0.092 g, 1.2 mmol) were suspended in ethanol (7 ml) and were refluxed for 5 h. After completion of the reaction, the reaction mixture was concentrated and poured into crushed ice. The precipitated product was filtered and washed with water. The resulting solid was recrystallized from a mixture of ethanol and water (*v*/*v* = 1:1), yield: 0.255 g (76.1%)

### Refinement

Carbon-bound H atoms were placed in calculated positions (C—H 0.95 Å for aromatic carbon atoms and C—H 1.00 Å for the methine group) and were included in the refinement in the riding model approximation, with *U*(H) set to 1.2*U*_eq_(C). The H atoms of the methyl groups were allowed to rotate with a fixed angle around the C—C bond to best fit the experimental electron density (HFIX 137 in the *SHELX* program suite (Sheldrick, 2008), with *U*(H) set to 1.5*U*_eq_(C). All nitrogen- and oxygen-bound H atoms were located on a difference Fourier map and refined freely.

### Crystal data

**Table:**

C_20_H_21_N_3_O_2_·H_2_O	*F*(000) = 752
*M**_r_* = 353.41	*D*_x_ = 1.284 Mg m^−^^3^
Monoclinic, *P*2_1_/*c*	Melting point = 428–430 K
Hall symbol: -P 2ybc	Mo *K*α radiation, λ = 0.71073 Å
*a* = 10.3516 (2) Å	Cell parameters from 8726 reflections
*b* = 12.4352 (3) Å	θ = 2.7–28.3°
*c* = 15.4101 (3) Å	µ = 0.09 mm^−^^1^
β = 112.798 (1)°	*T* = 200 K
*V* = 1828.68 (7) Å^3^	Block, yellow
*Z* = 4	0.41 × 0.34 × 0.18 mm

### Data collection

**Table:**

Bruker APEXII CCD diffractometer	4559 independent reflections
Radiation source: fine-focus sealed tube	3849 reflections with *I* > 2σ(*I*)
Graphite monochromator	*R*_int_ = 0.015
φ and ω scans	θ_max_ = 28.4°, θ_min_ = 2.1°
Absorption correction: multi-scan (*SADABS*; Bruker, 2008)	*h* = −13→13
*T*_min_ = 0.965, *T*_max_ = 0.984	*k* = −14→16
17882 measured reflections	*l* = −20→20

### Refinement

**Table:**

Refinement on *F*^2^	Primary atom site location: structure-invariant direct methods
Least-squares matrix: full	Secondary atom site location: difference Fourier map
*R*[*F*^2^ > 2σ(*F*^2^)] = 0.040	Hydrogen site location: inferred from neighbouring sites
*wR*(*F*^2^) = 0.114	H atoms treated by a mixture of independent and constrained refinement
*S* = 1.04	*w* = 1/[σ^2^(*F*_o_^2^) + (0.0577*P*)^2^ + 0.5735*P*] where *P* = (*F*_o_^2^ + 2*F*_c_^2^)/3
4559 reflections	(Δ/σ)_max_ < 0.001
255 parameters	Δρ_max_ = 0.30 e Å^−^^3^
0 restraints	Δρ_min_ = −0.21 e Å^−^^3^

### Fractional atomic coordinates and isotropic or equivalent isotropic displacement parameters (Å^2^)

**Table:**

	*x*	*y*	*z*	*U*_iso_*/*U*_eq_	
O1	−0.05984 (10)	0.17652 (9)	0.56849 (7)	0.0425 (2)	
O2	0.51356 (9)	0.21848 (9)	0.56375 (7)	0.0427 (2)	
O3	0.79358 (10)	0.22697 (7)	0.16165 (6)	0.0315 (2)	
N1	0.08635 (11)	0.45919 (8)	0.30709 (7)	0.0313 (2)	
N2	0.16757 (11)	0.51591 (8)	0.38313 (7)	0.0282 (2)	
N3	0.08322 (10)	0.08888 (8)	0.35769 (6)	0.0246 (2)	
C1	0.31972 (13)	0.50359 (9)	0.54994 (8)	0.0277 (2)	
C2	0.26550 (17)	0.58840 (11)	0.58442 (9)	0.0411 (3)	
H2	0.1723	0.6121	0.5501	0.049*	
C3	0.3462 (2)	0.63860 (12)	0.66841 (10)	0.0515 (4)	
H3A	0.3079	0.6960	0.6915	0.062*	
C4	0.4812 (2)	0.60560 (14)	0.71812 (10)	0.0546 (4)	
H4	0.5368	0.6403	0.7754	0.066*	
C5	0.53562 (17)	0.52211 (18)	0.68471 (11)	0.0608 (5)	
H5	0.6292	0.4994	0.7192	0.073*	
C6	0.45549 (14)	0.47036 (14)	0.60104 (10)	0.0439 (3)	
H6	0.4941	0.4122	0.5791	0.053*	
C7	0.23241 (11)	0.45289 (9)	0.45943 (8)	0.0240 (2)	
C8	0.19044 (11)	0.34797 (9)	0.43179 (7)	0.0216 (2)	
C9	0.09862 (13)	0.35805 (9)	0.33685 (8)	0.0285 (2)	
H9	0.0509	0.2992	0.2985	0.034*	
C10	0.22607 (10)	0.24440 (8)	0.48768 (7)	0.0204 (2)	
H10	0.2897	0.2617	0.5539	0.025*	
C11	0.30207 (11)	0.16707 (9)	0.44653 (7)	0.0219 (2)	
C12	0.22486 (11)	0.09714 (9)	0.37820 (7)	0.0233 (2)	
C13	0.02198 (11)	0.12349 (9)	0.41735 (7)	0.0232 (2)	
C14	0.09434 (11)	0.19160 (9)	0.48922 (7)	0.0221 (2)	
C15	0.04407 (12)	0.21902 (9)	0.56260 (8)	0.0262 (2)	
C16	0.12422 (15)	0.30098 (12)	0.63511 (9)	0.0382 (3)	
H16A	0.1371	0.3661	0.6035	0.057*	
H16B	0.2160	0.2715	0.6749	0.057*	
H16C	0.0718	0.3188	0.6742	0.057*	
C17	0.45534 (12)	0.17368 (9)	0.48711 (8)	0.0275 (2)	
C18	0.54636 (14)	0.12884 (13)	0.43963 (10)	0.0403 (3)	
H18A	0.5101	0.1522	0.3738	0.061*	
H18B	0.5457	0.0501	0.4423	0.061*	
H18C	0.6425	0.1550	0.4719	0.061*	
C19	−0.12248 (12)	0.07969 (11)	0.39365 (9)	0.0327 (3)	
H19A	−0.1567	0.0476	0.3307	0.049*	
H19B	−0.1853	0.1381	0.3949	0.049*	
H19C	−0.1199	0.0247	0.4399	0.049*	
C20	0.27302 (13)	0.02042 (10)	0.32129 (9)	0.0320 (3)	
H20A	0.1915	−0.0152	0.2742	0.048*	
H20B	0.3352	−0.0337	0.3631	0.048*	
H20C	0.3237	0.0603	0.2895	0.048*	
H3	0.0349 (17)	0.0442 (14)	0.3127 (12)	0.041 (4)*	
H2A	0.1834 (19)	0.5891 (15)	0.3761 (12)	0.052 (5)*	
H3B	0.708 (2)	0.2444 (15)	0.1288 (13)	0.052 (5)*	
H3C	0.8389 (19)	0.2649 (16)	0.1352 (12)	0.051 (5)*	

### Atomic displacement parameters (Å^2^)

**Table:**

	*U*^11^	*U*^22^	*U*^33^	*U*^12^	*U*^13^	*U*^23^
O1	0.0411 (5)	0.0483 (6)	0.0488 (6)	−0.0131 (4)	0.0291 (4)	−0.0137 (4)
O2	0.0226 (4)	0.0535 (6)	0.0444 (5)	0.0012 (4)	0.0046 (4)	−0.0123 (5)
O3	0.0288 (4)	0.0270 (5)	0.0379 (5)	0.0030 (3)	0.0118 (4)	0.0019 (3)
N1	0.0339 (5)	0.0258 (5)	0.0265 (5)	0.0009 (4)	0.0034 (4)	0.0030 (4)
N2	0.0321 (5)	0.0202 (5)	0.0278 (5)	−0.0003 (4)	0.0068 (4)	0.0030 (4)
N3	0.0256 (5)	0.0228 (5)	0.0229 (4)	−0.0016 (4)	0.0066 (4)	−0.0041 (3)
C1	0.0336 (6)	0.0220 (5)	0.0258 (5)	−0.0059 (4)	0.0098 (4)	0.0007 (4)
C2	0.0586 (9)	0.0276 (7)	0.0334 (6)	0.0049 (6)	0.0137 (6)	−0.0011 (5)
C3	0.0906 (13)	0.0282 (7)	0.0366 (7)	−0.0079 (7)	0.0256 (8)	−0.0070 (6)
C4	0.0712 (11)	0.0577 (10)	0.0309 (6)	−0.0320 (9)	0.0153 (7)	−0.0133 (7)
C5	0.0375 (8)	0.0912 (14)	0.0410 (8)	−0.0132 (8)	0.0014 (6)	−0.0141 (9)
C6	0.0315 (6)	0.0564 (9)	0.0376 (7)	−0.0012 (6)	0.0065 (5)	−0.0101 (6)
C7	0.0239 (5)	0.0216 (5)	0.0254 (5)	0.0004 (4)	0.0084 (4)	0.0013 (4)
C8	0.0214 (5)	0.0197 (5)	0.0227 (5)	0.0011 (4)	0.0072 (4)	0.0007 (4)
C9	0.0317 (6)	0.0227 (6)	0.0250 (5)	0.0000 (4)	0.0043 (4)	0.0006 (4)
C10	0.0202 (5)	0.0187 (5)	0.0204 (4)	0.0003 (4)	0.0055 (4)	−0.0001 (4)
C11	0.0225 (5)	0.0197 (5)	0.0234 (5)	0.0029 (4)	0.0087 (4)	0.0029 (4)
C12	0.0267 (5)	0.0196 (5)	0.0227 (5)	0.0046 (4)	0.0087 (4)	0.0034 (4)
C13	0.0230 (5)	0.0207 (5)	0.0247 (5)	0.0006 (4)	0.0080 (4)	0.0022 (4)
C14	0.0223 (5)	0.0205 (5)	0.0228 (5)	0.0008 (4)	0.0080 (4)	0.0012 (4)
C15	0.0273 (5)	0.0251 (6)	0.0271 (5)	0.0015 (4)	0.0116 (4)	−0.0003 (4)
C16	0.0427 (7)	0.0440 (8)	0.0337 (6)	−0.0104 (6)	0.0212 (5)	−0.0139 (5)
C17	0.0238 (5)	0.0244 (6)	0.0333 (6)	0.0033 (4)	0.0101 (4)	0.0046 (4)
C18	0.0289 (6)	0.0501 (8)	0.0464 (7)	0.0014 (6)	0.0195 (6)	−0.0014 (6)
C19	0.0261 (6)	0.0351 (7)	0.0352 (6)	−0.0075 (5)	0.0100 (5)	−0.0066 (5)
C20	0.0347 (6)	0.0287 (6)	0.0321 (6)	0.0065 (5)	0.0123 (5)	−0.0054 (5)

### Geometric parameters (Å, º)

**Table:**

O1—C15	1.2329 (15)	C8—C10	1.5133 (14)
O2—C17	1.2316 (15)	C9—H9	0.9500
O3—H3B	0.86 (2)	C10—C14	1.5219 (14)
O3—H3C	0.87 (2)	C10—C11	1.5272 (14)
N1—C9	1.3279 (16)	C10—H10	1.0000
N1—N2	1.3468 (14)	C11—C12	1.3608 (15)
N2—C7	1.3549 (14)	C11—C17	1.4649 (15)
N2—H2A	0.938 (19)	C12—C20	1.5055 (15)
N3—C13	1.3726 (14)	C13—C14	1.3646 (15)
N3—C12	1.3788 (15)	C13—C19	1.4977 (15)
N3—H3	0.878 (17)	C14—C15	1.4565 (15)
C1—C6	1.3814 (18)	C15—C16	1.5025 (16)
C1—C2	1.3931 (18)	C16—H16A	0.9800
C1—C7	1.4775 (15)	C16—H16B	0.9800
C2—C3	1.387 (2)	C16—H16C	0.9800
C2—H2	0.9500	C17—C18	1.5060 (17)
C3—C4	1.371 (3)	C18—H18A	0.9800
C3—H3A	0.9500	C18—H18B	0.9800
C4—C5	1.373 (3)	C18—H18C	0.9800
C4—H4	0.9500	C19—H19A	0.9800
C5—C6	1.391 (2)	C19—H19B	0.9800
C5—H5	0.9500	C19—H19C	0.9800
C6—H6	0.9500	C20—H20A	0.9800
C7—C8	1.3888 (15)	C20—H20B	0.9800
C8—C9	1.4079 (14)	C20—H20C	0.9800
			
H3B—O3—H3C	102.1 (16)	C12—C11—C10	118.75 (9)
C9—N1—N2	104.58 (9)	C17—C11—C10	115.75 (9)
N1—N2—C7	112.61 (10)	C11—C12—N3	118.60 (10)
N1—N2—H2A	119.0 (11)	C11—C12—C20	128.90 (10)
C7—N2—H2A	127.7 (11)	N3—C12—C20	112.46 (10)
C13—N3—C12	123.31 (9)	C14—C13—N3	119.18 (10)
C13—N3—H3	117.2 (11)	C14—C13—C19	127.29 (10)
C12—N3—H3	117.3 (11)	N3—C13—C19	113.53 (10)
C6—C1—C2	118.71 (12)	C13—C14—C15	121.77 (10)
C6—C1—C7	121.70 (11)	C13—C14—C10	117.93 (9)
C2—C1—C7	119.58 (11)	C15—C14—C10	120.19 (9)
C3—C2—C1	120.69 (15)	O1—C15—C14	122.76 (11)
C3—C2—H2	119.7	O1—C15—C16	118.82 (10)
C1—C2—H2	119.7	C14—C15—C16	118.41 (10)
C4—C3—C2	120.12 (15)	C15—C16—H16A	109.5
C4—C3—H3A	119.9	C15—C16—H16B	109.5
C2—C3—H3A	119.9	H16A—C16—H16B	109.5
C3—C4—C5	119.63 (13)	C15—C16—H16C	109.5
C3—C4—H4	120.2	H16A—C16—H16C	109.5
C5—C4—H4	120.2	H16B—C16—H16C	109.5
C4—C5—C6	120.89 (16)	O2—C17—C11	118.77 (11)
C4—C5—H5	119.6	O2—C17—C18	117.87 (11)
C6—C5—H5	119.6	C11—C17—C18	123.36 (11)
C1—C6—C5	119.96 (15)	C17—C18—H18A	109.5
C1—C6—H6	120.0	C17—C18—H18B	109.5
C5—C6—H6	120.0	H18A—C18—H18B	109.5
N2—C7—C8	106.64 (9)	C17—C18—H18C	109.5
N2—C7—C1	119.24 (10)	H18A—C18—H18C	109.5
C8—C7—C1	134.00 (10)	H18B—C18—H18C	109.5
C7—C8—C9	103.89 (9)	C13—C19—H19A	109.5
C7—C8—C10	130.29 (9)	C13—C19—H19B	109.5
C9—C8—C10	125.78 (10)	H19A—C19—H19B	109.5
N1—C9—C8	112.27 (10)	C13—C19—H19C	109.5
N1—C9—H9	123.9	H19A—C19—H19C	109.5
C8—C9—H9	123.9	H19B—C19—H19C	109.5
C8—C10—C14	110.72 (8)	C12—C20—H20A	109.5
C8—C10—C11	110.53 (8)	C12—C20—H20B	109.5
C14—C10—C11	110.10 (8)	H20A—C20—H20B	109.5
C8—C10—H10	108.5	C12—C20—H20C	109.5
C14—C10—H10	108.5	H20A—C20—H20C	109.5
C11—C10—H10	108.5	H20B—C20—H20C	109.5
C12—C11—C17	125.48 (10)		
			
C9—N1—N2—C7	1.05 (14)	C14—C10—C11—C12	33.04 (13)
C6—C1—C2—C3	0.0 (2)	C8—C10—C11—C17	91.88 (11)
C7—C1—C2—C3	−178.86 (13)	C14—C10—C11—C17	−145.49 (9)
C1—C2—C3—C4	0.5 (2)	C17—C11—C12—N3	169.86 (10)
C2—C3—C4—C5	−0.5 (2)	C10—C11—C12—N3	−8.51 (15)
C3—C4—C5—C6	−0.1 (3)	C17—C11—C12—C20	−7.34 (18)
C2—C1—C6—C5	−0.6 (2)	C10—C11—C12—C20	174.29 (10)
C7—C1—C6—C5	178.25 (14)	C13—N3—C12—C11	−18.40 (16)
C4—C5—C6—C1	0.7 (3)	C13—N3—C12—C20	159.25 (10)
N1—N2—C7—C8	−0.31 (13)	C12—N3—C13—C14	16.24 (16)
N1—N2—C7—C1	−176.91 (10)	C12—N3—C13—C19	−163.95 (10)
C6—C1—C7—N2	−128.63 (13)	N3—C13—C14—C15	−171.08 (10)
C2—C1—C7—N2	50.24 (16)	C19—C13—C14—C15	9.13 (18)
C6—C1—C7—C8	55.9 (2)	N3—C13—C14—C10	12.63 (15)
C2—C1—C7—C8	−125.23 (15)	C19—C13—C14—C10	−167.15 (11)
N2—C7—C8—C9	−0.53 (12)	C8—C10—C14—C13	87.54 (12)
C1—C7—C8—C9	175.35 (13)	C11—C10—C14—C13	−34.99 (13)
N2—C7—C8—C10	−178.25 (10)	C8—C10—C14—C15	−88.80 (11)
C1—C7—C8—C10	−2.4 (2)	C11—C10—C14—C15	148.67 (10)
N2—N1—C9—C8	−1.40 (14)	C13—C14—C15—O1	5.77 (18)
C7—C8—C9—N1	1.23 (14)	C10—C14—C15—O1	−178.02 (11)
C10—C8—C9—N1	179.10 (10)	C13—C14—C15—C16	−175.47 (11)
C7—C8—C10—C14	119.14 (12)	C10—C14—C15—C16	0.73 (16)
C9—C8—C10—C14	−58.14 (14)	C12—C11—C17—O2	−160.83 (12)
C7—C8—C10—C11	−118.59 (12)	C10—C11—C17—O2	17.58 (16)
C9—C8—C10—C11	64.13 (14)	C12—C11—C17—C18	19.51 (18)
C8—C10—C11—C12	−89.60 (11)	C10—C11—C17—C18	−162.08 (11)

### Hydrogen-bond geometry (Å, º)

**Table:**

*D*—H···*A*	*D*—H	H···*A*	*D*···*A*	*D*—H···*A*
N2—H2*A*···O3^i^	0.938 (19)	1.855 (19)	2.7822 (14)	169.1 (16)
N3—H3···N1^ii^	0.878 (17)	2.075 (17)	2.9454 (13)	171.1 (15)
O3—H3*B*···O2^iii^	0.86 (2)	1.92 (2)	2.7838 (13)	175.8 (17)
O3—H3*C*···O1^iv^	0.87 (2)	1.88 (2)	2.7372 (14)	169.8 (18)
C6—H6···O2	0.95	2.44	3.282 (2)	148
C10—H10···O2	1.00	2.33	2.7624 (13)	105

Symmetry codes: (i) −*x*+1, *y*+1/2, −*z*+1/2; (ii) −*x*, *y*−1/2, −*z*+1/2; (iii) *x*, −*y*+1/2, *z*−1/2; (iv) *x*+1, −*y*+1/2, *z*−1/2.
